# Perfringolysin O: The Underrated *Clostridium perfringens* Toxin?

**DOI:** 10.3390/toxins7051702

**Published:** 2015-05-14

**Authors:** Stefanie Verherstraeten, Evy Goossens, Bonnie Valgaeren, Bart Pardon, Leen Timbermont, Freddy Haesebrouck, Richard Ducatelle, Piet Deprez, Kristin R. Wade, Rodney Tweten, Filip Van Immerseel

**Affiliations:** 1 Department of Pathology, Bacteriology and Avian Diseases, Faculty of Veterinary Medicine, Ghent University, Salisburylaan 133, 9820 Merelbeke, Belgium; E-Mails: s.verherstraeten@gmail.com (S.V.); evy.goossens@ugent.be (E.G.); leen.timbermont@ugent.be (L.T.); freddy.haesebrouck@ugent.be (F.H.); richard.ducatelle@ugent.be (R.D.); 2 Department of Internal Medicine and Clinical Biology of Large Animals, Faculty of Veterinary Medicine, Ghent University, Salisburylaan 133, 9820 Merelbeke, Belgium; E-Mails: bonnie.valgaeren@ugent.be (B.V.); bart.pardon@ugent.be (B.P.); piet.deprez@ugent.be (P.D.); 3 Department of Microbiology and Immunology, University of Oklahoma Health Sciences Center, Oklahoma City, OK 73104, USA; E-Mails: kristin-wade@ouhsc.edu (K.R.W.); rodney-tweten@ouhsc.edu (R.T.)

**Keywords:** cholesterol-dependent cytolysin, gas gangrene, myonecrosis, necrohemorrhagic enteritis, enterotoxaemia, calves

## Abstract

The anaerobic bacterium *Clostridium perfringens* expresses multiple toxins that promote disease development in both humans and animals. One such toxin is perfringolysin O (PFO, classically referred to as θ toxin), a pore-forming cholesterol-dependent cytolysin (CDC). PFO is secreted as a water-soluble monomer that recognizes and binds membranes via cholesterol. Membrane-bound monomers undergo structural changes that culminate in the formation of an oligomerized prepore complex on the membrane surface. The prepore then undergoes conversion into the bilayer-spanning pore measuring approximately 250–300 Å in diameter. PFO is expressed in nearly all identified *C. perfringens* strains and harbors interesting traits that suggest a potential undefined role for PFO in disease development. Research has demonstrated a role for PFO in gas gangrene progression and bovine necrohemorrhagic enteritis, but there is limited data available to determine if PFO also functions in additional disease presentations caused by *C. perfringens*. This review summarizes the known structural and functional characteristics of PFO, while highlighting recent insights into the potential contributions of PFO to disease pathogenesis.

## 1. Introduction

*Clostridium perfringens* is an anaerobic, spore-forming Gram-positive bacterium often found as a normal inhabitant of animal and human intestines [1,2,3]. However, by mechanisms and stimuli that are not fully understood, *C. perfringens* undergoes rapid proliferation, while producing several toxins, resulting in disease onset. Classification of *C. perfringens* strains is based on the production of α, β, ε and ι toxins, considered the four major clostridial toxins. Additional toxins are also expressed and secreted by *C. perfringens*, such as the pore-forming toxin perfringolysin O (PFO), formerly referred to as θ toxin [4,5]. PFO, in synergy with α toxin, is involved in the development of gas gangrene and necrohemorrhagic enteritis in calves [6,7]. PFO might also be important in other diseases caused by *C. perfringens*, since PFO is known to potentiate the lethal effect of ε toxin in a mouse model for type D enterotoxaemia, a naturally occurring disease in sheep and goats [8]. The PFO pore-forming mechanism has been well studied, leading to several insights into the PFO structure and function. However, the role of PFO in disease development is not often evaluated in pathogenesis models, despite the fact that PFO harbors characteristics that suggest a more important role in animal and human diseases than previously thought. We present herein an overview of the known characteristics of PFO and hypothesize about PFO contributions to the pathology of animal and human diseases.

PFO belongs to the cholesterol-dependent cytolysin (CDC) family, which acts as pore-forming toxins on cholesterol-containing membranes [9]. Similar toxins have been identified in *Streptococcus*, *Bacillus*, *Listeria* and many other genera. These CDCs share a high degree of primary structural homology. PFO is viewed as the archetype CDC, and thus, data presented in this review for PFO can be partially extrapolated to other CDCs and provide the basis for a general CDC pore-forming mechanism [10].

## 2. Genetics

The genome of *Clostridium perfringens* consists of a single circular chromosome and additional extra-chromosomal plasmids. Many of the toxins produced are plasmid-encoded, including β, ε and ι toxins, while the genes encoding PFO (*pfoA*) and α toxin (*plc*) are located on the chromosome [11,12,13,14,15,16,17,18]. The *pfoA* gene is suspected to be encoded by nearly all *C. perfringens* strains, although genome comparisons revealed that the majority of the enterotoxin-producing food poisoning strains lack *pfoA* [16,17,19,20]. The structural gene *pfoA* has been cloned, sequenced and mapped [21,22,23]. The primary protein structure derived from the nucleotide sequence includes 500 amino acid residues and a 27-residue signal peptide [24]. Based on these data, Tweten [24] predicted a molecular weight of 52,469 daltons (Da) for PFO. However, variations occur in the primary structure and in the PFO chromosomal location [19,24,25]. Recombination presumably explains the variations in the location and sequence of *pfoA* and other chromosome-encoded virulence genes, although the primary structure of PFO is well conserved [11,25,26]. The most conserved region of *pfoA* surrounds the undecapeptide, a tryptophan-rich loop that contains three tryptophan residues and the only cysteine residue in secreted PFO [10,24].

## 3. PFO Structure

**Figure 1:**
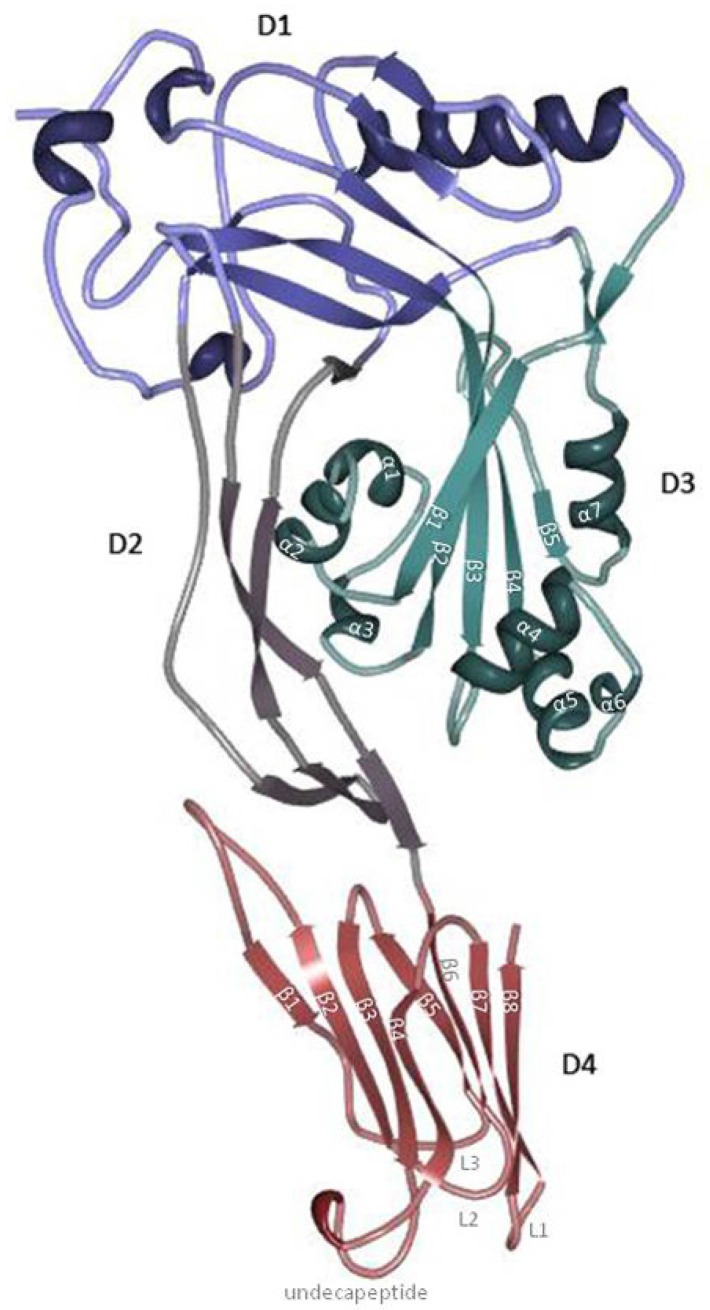
Perfringolysin O’s (PFO) structure. PFO is dominated by β-strands and is divided into four domains. Domain 4 (red; D4) consists of two β-sheets of four β-strands (D4 β1–4 and D4 β5–8) packed together in a β-sandwich structure connected by four loops (L1, L2, L3 and undecapeptide). Domain 3 (green; D3) contains one core β-sheet (D3 β1–5) flanked by two sets of three α-helices (D3 α1–3 and D3 α4–6) and an additional α-helix (α7) that connects β5 with domain 1 (blue; D1). Domain 1 and domain 2 (purple; D2) connect D3 and D4. D2 is elongated and contains a β-sheet. D1 consists of a β-sheet and four α-helices. The figure was made with RCSB PDB Protein Workshop 4.1.0 (RCSB Protein Data Bank, Piscataway, NJ and La Jolla, CA, USA, 2014) and adapted with Adobe Photoshop CS3 extended (Adobe Systems Incorporated, San Jose, CA, USA, 2007).

**Figure 2:**
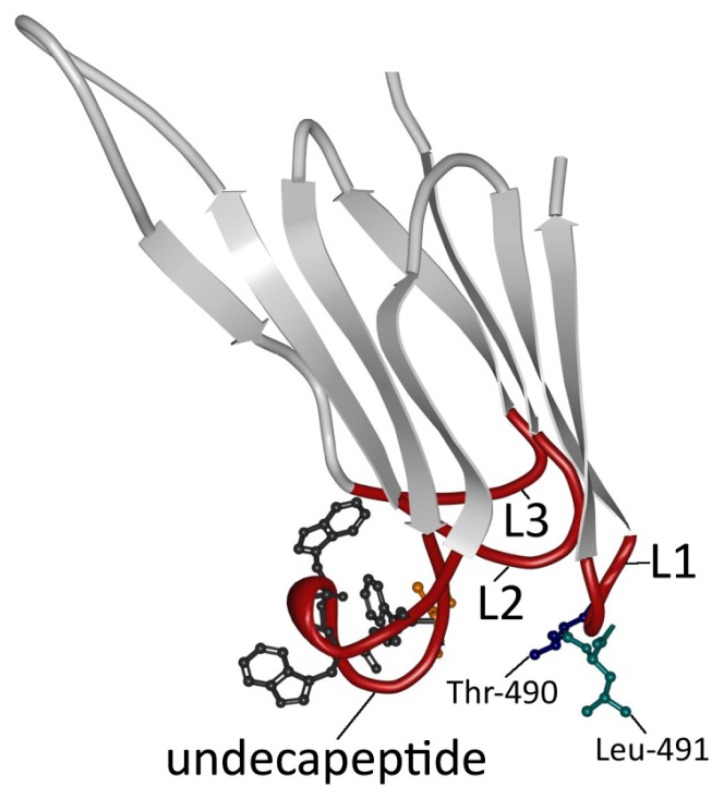
Detailed view of PFO domain 4. Domain 4 (D4) consists of two β-sheets of four β-strands (light grey; D4 β1–4 and D4 β5–8) connected by four loops (red; L1, L2, L3 and undecapeptide). The undecapeptide contains three out of six tryptophans (dark grey) in PFO and the only cysteine (orange) present in the secreted form. Recent results have shown that only the threonine (blue) and the leucine (green) in L1 are essential for the recognition and binding of the membrane [49]. The figure was made with RCSB PDB Protein Workshop 4.1.0 (RCSB Protein Data Bank, Piscataway, NJ and La Jolla, CA, USA, 2014) and adapted with Adobe Photoshop CS3 extended (Adobe Systems Incorporated, San Jose, CA, USA, 2007).

PFO contains a typical signal peptide that facilitates its secretion by the general secretory pathway (GSP), which results in an extracellular water-soluble monomer [10,24]. This signal peptide is recognized by the GSP and is cleaved upon passage through the cell membrane [27,28]. Solovyova *et al.* [29] hypothesized that PFO forms dimers in solution at high concentrations, and the crystals of PFO exhibited a head-to-tail dimer [30]. Whether PFO forms dimers at physiological concentration remains unclear. The solved crystal structure reveals that PFO monomers have an elongated structure divided into four domains that are dominated by β-strands (Figure 1) [30]. Domain 4 (D4) contains two β-sheets, each consisting of four β-strands (D4 β1–4 and D4 β5–8) packed together in a β-sandwich structure connected by four loops (L1, L2, L3 and undecapeptide) (Figure 1 and Figure 2) [30,31]. Domain 3 (D3) consists of one core β-sheet (D3 β1–5) flanked by two sets of three α-helices (D3 α1–3 and D3 α4–6) (Figure 1; see also Figure 4a) [30]. An additionalα-helix (α7) connects β5 with domain 1 (D1). Domain 1 and 2 (D2) connect D3 and D4 (Figure 1). The elongated D2 contains a β-sheet, whereas D1 consists of a β-sheet and four α-helices (Figure 1) [30].

In the membrane-embedded pore complex, the β-strands of D3 from several monomers form a single closed cylindrical β-sheet, forming a β-barrel. The concerted action of at least 35 monomers is required to create a large transmembrane β-barrel of 250–300 Å in diameter [30,32,33,34].

## 4. Membrane Binding

A conserved feature of CDCs is the requirement for cholesterol, a common constituent of mammalian membranes, for their cytolytic activity [9,35]. Therefore, cholesterol was long suspected to be the receptor for the CDCs, because cholesterol is essential for pore formation, and the activity of CDCs is inhibited when CDCs are pre-exposed to cholesterol [35,36,37,38,39,40]. Results on intermedilysin (ILY), a CDC produced by *Streptococcus intermedius*, indicate that not all CDCs utilize cholesterol for binding [41,42]. Nevertheless, cholesterol remains crucial for pore formation in these CDCs.

The cytolytic mechanism of PFO begins with D4-mediated binding to the plasma membrane of the eukaryotic cell [9,31,33,43]. D4 interacts with the membrane via loops at the tip of the domain that connect the two β-sheets (Figure 2). The rest of D4 does not contact the membrane surface [31,33]. One of these loops corresponds to the conserved undecapeptide (Figure 2). Until relatively recently, it was thought that this tryptophan-rich loop functioned as the cholesterol binding motif, since modification of this region influenced PFO binding and pore-forming activity [44,45,46,47]. Furthermore, the cysteine in the undecapeptide seemed to be involved in the cytolytic mechanism, since mutation and oxidation of this cysteine also altered the conformation of the tryptophan-rich loop and hindered membrane binding (Figure 2) [48]. However, more recent evidence shows that the three other loops, L1–L3, are important in cholesterol recognition and binding rather than the undecapeptide (Figure 2) [47]. The undecapeptide, on the other hand, contributes to both the anchoring of PFO to the membrane and to the allosteric coupling of binding to distal conformational and structural changes necessary for the insertion of the β-barrel in the membrane, which explains why mutations of these tryptophans influence the cytotoxic activity [47,49,50]. In addition, cysteine modification prevents the prepore to pore transition (molecular mechanism of pore formation below) by influencing the tryptophan-rich loop [47]. The cholesterol binding motif was subsequently identified by Farrand *et al.* [51], who showed that only two residues, threonine-490 and leucine-491 in L1, are crucial in the recognition and binding of cholesterol in the membrane (Figure 2). The Thr-Leu pair is collectively termed the cholesterol-recognition motif, or CRM. Following binding initiation by the CRM, the L2 and L3 of D4 insert into the membrane and stabilize binding, thereby orientating PFO perpendicular to the membrane [31,34,52].

Interestingly, the CRM motif is conserved in all known CDCs, even in intermedilysin (ILY), which utilizes the human GPI-anchored CD59 (huCD59) as a membrane binding receptor, rather than cholesterol [30,31,43]. Following the ILY binding interaction with huCD59, ILY uses the CRM motif and L2 and L3 in exactly the same manner as PFO to form a cholesterol-dependent contact. ILY oligomerizes into the prepore intermediate, while bound to huCD59, but disengages from huCD59 during conversion to the pore. Thus, ILY cholesterol binding serves to anchor the ILY pore to the membrane following huCD59 disengagement [51,53].

**Figure 3:**
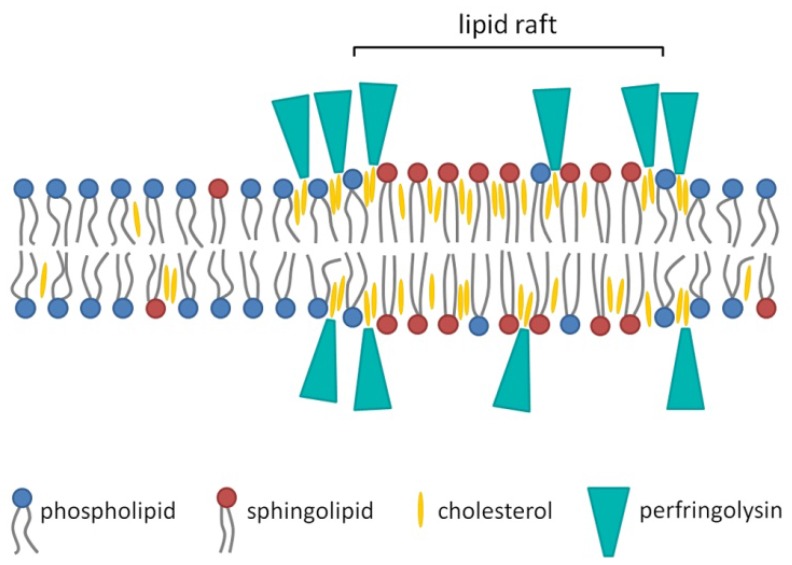
Schematic diagram of the association of PFO with the membrane bilayer. The binding of PFO to the membrane is not merely associated with sphingolipid- and cholesterol-rich liquid ordered membrane domains or lipid rafts. The association is highest and strongest on the edges of the ordered membrane domain, where cholesterol seems more accessible for PFO [59]. The figure was made with Microsoft Office PowerPoint 2007 (Microsoft Corporation, Redmond, WA, USA, 2007).

Because PFO only binds to membranes that contain a substantial amount of cholesterol, it was proposed that the binding was associated with the sphingolipid- and cholesterol-rich liquid ordered membrane domains or lipid rafts (Figure 3) [43,54,55,56,57]. This hypothesis was contradicted by Nelson *et al.* [58] and Flanagan *et al.* [36], who did not find an association between the presence of membrane rafts and the ability of PFO to bind. However, studies have demonstrated that the binding capacity of PFO depends on the accessibility of the cholesterol, which is influenced by packing of the phospholipid headgroups and the saturation of the cholesterol-phospholipid acyl chain, both of which can affect how tightly they pack with cholesterol [36,55,58]. More recent research showed that PFO has some affinity for lipid rafts, but the association is highest and strongest at the borders of the liquid ordered and liquid disordered membrane domains, where cholesterol seems more accessible to PFO (Figure 3) [59]. The binding of PFO is therefore not merely controlled by the amount of cholesterol present, but also by the lipid environment of the cholesterol. The thickness of the membrane bilayer may also affect the efficiency of pore formation, as modifying the length of the TMH can hinder its insertion into bilayers of various widths [60]. This is not altogether surprising, as the TMH length in the wild-type toxin should be optimal for TMH insertion into the targeted lipid environment surrounding membrane cholesterol.

## 5. Molecular Mechanism of Pore Formation

**Figure 4:**
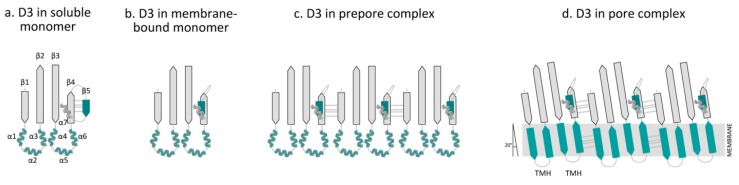
Overview of the conformational changes of domain 3 in the transition from a water-soluble monomer to the pore complex. Domain 3 (D3) contains a β-sheet (D3 β1–5) flanked by two sets of three α-helices (D3 α1–3 and α4–6) and an additional α-helix (α7) that connects β5 with domain 1 (D1). In the water-soluble monomer, β4 forms hydrogen bonds with β-strand β5 ((**a**) D3 in the soluble monomer). D4 binding to the membrane elicits conformational changes in D3, which causes the rotation of β5 away from the core β-sheet, thus exposing the edge of β4 ((**b**) D3 in the membrane-bound monomer). As a result, β4 can form contacts with an always-exposed β1 in the core β-sheet of an adjacent monomer ((**c**) D3 in the prepore complex). Upon conversion of the prepore to the pore, the two α-helical bundles refold into two amphipathic transmembrane β-hairpins (TMHs) ((**d**) D3 in the pore complex). In the pore complex, the β-strands of D3 monomers are tilted 20 degrees perpendicular to the membrane with a right-hand twist. The figure was made with Graphisoft ArchiCAD 13 (Graphisoft, Budapest, Central Hungary, Hungary, 2009).

**Figure 5:**
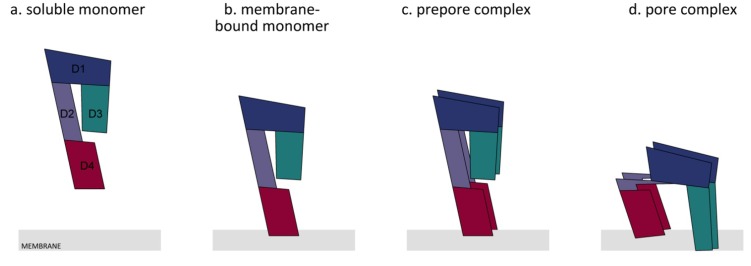
Overview of the general mechanism of the transition from a water-soluble monomer to the pore complex. PFO is secreted as a water-soluble monomer ((**a**) water-soluble monomer). Upon encountering a cholesterol-containing membrane, D4 (red) interacts with the membrane at the tip of the domain ((**b**) membrane-bound monomer). This triggers a conformational change in domain 3 (green; D3); thereby, D3 of adjacent monomers can interact with each other, and this causes the formation of a prepore complex ((**c**) prepore complex). The prepore complex is inserted into the membrane bilayer ((**d**) pore complex). A collapse of the PFO molecule occurs, which moves domains 3 and 1 (blue; D1) closer to the membrane. This is caused by a rotation of D4 that triggers the bending of domain 2 (purple; D2). The latter reduces the strength of the binding between D3 and D2, and thereby, D3 and D1 can move towards the membrane. The figure was made with Graphisoft ArchiCAD 13 (Graphisoft, Budapest, Central Hungary, Hungary, 2009).

Within the D3 core β-sheet, β-strand 4 (β4) forms four hydrogen bonds with β-strand 5 (β5), which prevents the association of β4 with β-strand 1 (β1) from an adjacent monomer (Figure 4a). The β4–β5 interaction prevents the spontaneous oligomerization of soluble monomers [9,61]. The binding of D4 to the membrane is coupled to conformational changes in D3, causing β5 to rotate around a conserved glycine-glycine pivot away from the core β-sheet (Figure 4b). As a result, β4 becomes exposed and can associate with the always-exposed β1 in the core β-sheet from an adjacent monomer (Figure 4c and Figure 5c) [61,62,63].

In the water-soluble monomer, the core β-sheet of D3 is flanked by two sets of α-helices (D3 α1–3 and D3 α4–6), which minimize the exposure of the hydrophobic side chains (Figure 4a) [31,64,65]. As a result of D4 binding to the membrane, conformational changes in D3 are induced that promote the formation of the prepore structure [61,63]. The prepore complex is composed of oligomerized monomers that have yet to insert their β-barrel (Figure 4c, Figure 5c) [66]. After formation of the prepore, the two α-helices unfurl into two amphipathic transmembrane β-hairpins (TMH1 and TMH2), which insert into the membrane in the pore complex (Figure 4d, Figure 5d) [64,65,67]. At first, these β-hairpins extend from the core β-sheet and move dynamically [33,52,62,68]. In the pore complex, the TMHs form backbone hydrogen bonds with TMHs of adjacent molecules to form the contiguous β-barrel (Figure 4d, Figure 5d). All monomers contribute two TMHs that are inserted into the membrane bilayer in a concerted, synchronized fashion to form the β-barrel [62,66,68]. TMH-synchronized insertion is energetically more favorable than the domino-type insertion of TMHs from individual monomers [69,70]. The TMHs completely span the membrane, with the hydrophobic surfaces facing the lipid core of the membrane and the hydrophilic surfaces exposed to the aqueous pore [65,67].

In the pore complex, the individual β-strands of D3 are tilted 20° perpendicular to the membrane with a right-hand twist (Figure 4d) [62]. A rotation of the D3 core β-sheet relative to D4 is needed to untwist the core β-sheet and align the TMHs (Figure 5d). D3 does not directly contact D4, but forms an interface with both D1 and D2 that connects D3 with D4 (Figure 1, Figure 5b) [30,31,43]. The stability of D1 increases upon membrane binding, which may be important for disengagement of the D2,3 interface [71]. When D1 is destabilized by the substitution of a tryptophan residue (Trp-165 in PFO), the interface between D2,3 fails to disengage [63,71]. Additionally, Wade *et al.* [72] recently showed that the rotation of β5 away from the core β-sheet in D3 allows the formation of a strong intermolecular electrostatic interaction, which drives the transition from the prepore to the pore by providing the free energy necessary to disrupt the interface between D3 and D1,2. These studies highlight the importance of the domain stability and the interactions between intra- and inter-domains for pore formation.

Czajkowsky *et al.* [34] and Ramachandran *et al.* [52] showed that D1 and D3 both undergo a 35–40 Å vertical collapse towards the membrane, suggesting that a conformational change must occur in D2 to facilitate this collapse. Such a conformational change is consistent with the bulge on the outer surface of the membrane-inserted oligomeric complex shown in a 3D reconstruction of the pneumolysin (PLY) pore complex, a CDC produced by *Streptococcus pneumoniae* [73]. Additionally, crystallographic modeling predicts that D4 undergoes a rotation relative to D2 that breaks several contacts between D2 and D3, thereby allowing the α-helical bundle to unfold and then refold into TMH (Figure 5d) [64]. Figure 5 summarizes the sequence of the general mechanism of pore complex formation by PFO.

## 6. Genetic Regulation

A quorum-sensing system homologous to the accessory gene regulator system (*agr*) of *Staphylococcus aureus* was found in *C. perfringens*, which could regulate the expression of several virulence factors in response to external stimuli [74,75,76]. Ohtani *et al.* [71] and Vidal *et al.* [76] showed that the expression of PFO and α toxin are regulated by the *agr* system, as the mutation of the *agr* locus influenced the early log-phase expression of both toxins. *C. perfringens* also harbors an additional quorum-sensing system homologous to the *luxS* system [77]. A *luxS* mutant has a reduced expression of PFO at the mid-exponential growth phase [77]. Additionally, the two-component signal transduction system consisting of the VirS sensor protein and the gene response regulator VirR regulates the expression of PFO, α toxin, collagenase and several housekeeping genes [78,79,80,81]. It is not yet completely clear if the *agr* and *luxS* system are coupled to the VirR/VirS system or by which stimuli the VirR/VirS system is activated. The signaling molecules and the mode of action remain to be elucidated [75,76,82].

The activation of VirS causes phosphorylation of VirR, allowing VirR to bind two VirR boxes, *i.e*., 12-bp repeated sequences located upstream of the *pfo* gene encoding PFO, which causes a direct regulation of *pfoA* transcription [75,80,83,84,85]. The expression of the PFO structural gene *pfoA* is abolished after mutation of *VirR* or *VirS* [79,80,81,84]. Shimizu *et al.* [22] suspected that the upstream *pfoR* gene coded for a regulatory protein involved in the expression of the PFO structural gene. Awad and Rood [86] contradicted this by showing that mutating *pfoR* did not influence *pfoA* expression. They hypothesized that under certain experimental conditions, the *pfoR* gene could have a regulatory role, such as in an infected lesion, but this has not been demonstrated.

## 7. The Role of PFO in Disease

*Clostridium perfringens* is the most important causative agent of clostridial myonecrosis (also called gas gangrene) [6,87]. This disease can arise when the anaerobic bacterium is introduced into muscle tissue, often as a consequence of a traumatic injury [88,89,90]. Clostridial myonecrosis is reported in several animal species, such as dogs, cats, cattle, sheep, goats and horses, but also occurs in humans [90]. The disease is characterized by rapid spreading of tissue necrosis within the muscle (thus the term myonecrosis), which can lead to death caused by systemic toxemia and shock, with very high mortality rates, if not promptly treated [88,89]. It is widely accepted that α toxin is an essential causative toxin involved in gas gangrene, because only strains producing α toxin are able to induce the typical pathology of myonecrosis with thrombosis in a mouse myonecrosis model [91,92,93]. The role of PFO in gas gangrene is less well understood. It appears to work synergistically with α toxin to effect peripheral to myonecrosis leukostasis and intravascular coagulopathy, whereas the majority of the myonecrosis can be attributed to α toxin alone [6,18,93,94,95,96]. Decreased mortality was observed after immunization with PFO prior to intramuscular injection of *C. perfringens* in mice [95,97]. Awad *et al.* [6] then demonstrated that PFO acts in synergy with α toxin to cause gas gangrene, as virulence of a mutant lacking both genes was drastically decreased in a mouse myonecrosis model. Furthermore, complementation with both toxins could produce a more severe pathology than the strain complemented with α toxin alone. These results clearly suggest that PFO and α toxin are involved in the pathology of clostridial myonecrosis or gas gangrene. However, the individual contributions of both toxins to disease are not fully defined.

Vascular leukostasis and the paucity of leukocyte infiltration to the site of infection are two characteristics of gas gangrene [6,93]. Intramuscular injection of a crude clostridial toxin preparation triggers the formation of intravascular aggregates consisting of platelets, which initially move freely within blood vessels [93,98]. Fibrin and leukocytes are rapidly added to the aggregate, thus trapping the aggregates and obstructing the blood vessels. The thrombi reduce or even completely stop the local blood flow [98,99]. This process impairs the movement of inflammatory cells to the infected tissue, which could explain the lack of phagocytic cells at the later stages of a gas gangrene infection. α toxin is shown to directly activate gpIIbIIIa, a receptor on platelets involved in platelet aggregation [100,101,102]. PFO causes an augmented expression of adhesion molecules, such as CD11b/CD18 on leukocytes and intracellular adhesion molecule 1 (ICAM-1) and platelet-activating factor on human endothelial cells [94,97,103,104,105]. The increased expression of platelet-activating factor is linked to leukocyte adhesion to human endothelial cells after exposure to α toxin [99,102,106]. Therefore, the increased expression of the platelet and endothelial cell adhesion factors, as well as chemokines might also contribute to the adhesion of platelet-leukocyte aggregates to endothelial cells lining the blood vessel, which contributes to thrombosis and subsequent reduction in blood flow [100,102].

This strong leukocyte adhesion to endothelial cells also impairs the transendothelial migration of leukocytes and subsequent infiltration of the infection site [94,97,102,107]. This is of benefit for *C. perfringens* residing in the tissue, as this simultaneously promotes the anaerobic conditions of the tissue and reduces inflammation [18]. Moreover, a direct cytotoxic effect of PFO and α toxin on endothelial cells has been demonstrated, which likely also impairs leukocyte transmigration [99,107]. This disruption of the endothelium also contributes to progressive edema [107]. Furthermore, the lack of acute inflammatory cells in the infected tissue could also be due to a direct suppression of the immune response by PFO and α toxin, as a direct cytolytic effect on leukocytes has been observed in the presence of a high concentration of PFO or α toxin [108].

While the later stages of a myonecrosis infection are characterized by a paucity of phagocytic cells, the presence of phagocytic cells could be of importance to control the bacteria at the onset of infection, when there are few bacteria present in the tissue. *C. perfringens* is able to survive in the presence of macrophages and can escape the phagosome [109]. Successful phagosome escape is only accomplished in the presence of both α toxin and PFO. Furthermore, PFO is the most important factor for macrophage cytotoxicity [110].

In summary, PFO causes macrophage cytotoxicity in the early stages of myonecrosis and is important for thrombi formation in the later stages of infection. The latter is caused by effects on the expression of adhesion factors and chemokines by endothelial cells and leukocytes. Although PFO is involved, α toxin is clearly also involved, suggesting these two toxins act synergistically.

Uzal *et al.* [18] suggested that PFO is not a main causative toxin for most intestinal diseases caused by *C. perfringens*, as type C strains lacking the *pfoA* and α toxin gene caused lesions comparable to natural cases in a rabbit ileal loop model, and the *pfoA* gene is absent in the genome of most enterotoxin-producing food poisoning strains [16,17,19,20,111]. Recently, however, the involvement of PFO in the pathogenesis of bovine necrohemorrhagic enteritis or enterotoxaemia has been demonstrated [7]. This disease is most typically characterized by sudden death with macroscopic post-mortem identification of necrotic and hemorrhagic lesions in the small intestine [4,112]. Necrohemorrhagic enteritis is a major cause of mortality in veal calves, causing important economic losses [113,114,115]. Comparable to the data on myonecrosis, PFO acts synergistically with α toxin in the development of this disease, since a mutant lacking both genes had a decreased ability to induce necrohemorrhagic lesions and only complementation with both genes restored activity to wild-type levels [7].

While gas gangrene is characterized by tissue necrosis, thrombosis formation and a lack of leukocyte infiltration at the site of infection, bovine necrohemorrhagic enteritis is associated with capillary congestion, hemorrhages and inflammation [116,117,118]. PFO and α toxin are involved in both gas gangrene and necrohemorrhagic enteritis, but the different phenotypes suggest that these toxins may act in different ways in the disease pathology. Both toxins have a strong cytotoxic effect on bovine endothelial cells, which might indicate that these toxins act by targeting the endothelium, potentially explaining capillary hemorrhages [7].

Intravenous injection of recombinant PFO in rabbits causes an increase in cardiac output [95,96]. This is not due to a direct cardiotoxic effect, but rather to a reduction in vascular resistance and subsequent vasodilatation [6,95,96]. Relaxation of the blood vessels is proposed to result as an indirect effect of PFO on endogenous mediators, such as cytokines [95,96]. After injection of recombinant α toxin in rabbits, however, the vascular resistance was maintained while a reduction in cardiac output and arterial pressure were observed [95,96,99,119]. This was attributed to a direct effect of α toxin on the myocardial contractility [95,96]. Rabbits inoculated intravenously with crude *C. perfringens* supernatant exhibit an initial increase in blood flow, linked to the reduction in vascular resistance due to PFO [96]. However, a reduction in cardiac output and arterial pressure are observed afterwards, which are attributed to α toxin [96,99,119]. These results, combined with the pathological observations of bovine necrohemorrhagic enteritis (*i.e*., inflammation and hemorrhages) and loss of vascular integrity, seem to be in accordance with the role of PFO in this disease. On the contrary, hemorrhages are not observed in myonecrosis. Moreover, gas gangrene does not appear to be associated with a reduction in vascular resistance, as during an amputation of an infected limb/area, removal of all non-bleeding tissue is recommended [98]. Intramuscular injection of a crude clostridial toxin preparation in rodents triggers the formation of intravascular aggregates, leading to thrombi formation [93,98]. No intravascular aggregates and subsequent thrombosis are observed in bovine necrohemorrhagic enteritis, which partially explains the inflammation present in bovine necrohemorrhagic enteritis, which is in contrast to the lack of leukocyte infiltration (and subsequent inflammation) in gas gangrene. These observations indicate that the effects exerted by both PFO and α toxin are species and target organ dependent. In advanced stages of disease, myonecrosis develops further into systemic toxemia and multi-organ failure caused by cardiovascular collapse as a result of the effects on systemic blood pressure and cardiac function linked with α toxin [87,107]. Bovine necrohemorrhagic enteritis is characterized by sudden death, which might also be due to a direct effect of α toxin on myocardial contractility. However, more research is needed to explain the observed differences in pathology between gas gangrene and bovine necrohemorrhagic enteritis. Further insights into the mode of action of these toxins are needed to unravel their effects on different target organs and identify the relative contribution of both toxins to pathogenesis.

The possible role of PFO in other diseases caused by *C. perfringens* needs to be more clearly defined. Several clostridial disease phenotypes are thought to result from a specific causative toxin. However, PFO could be of importance in these diseases, as well. For example, recently, Fernandez-Miyakawa *et al.* [8] demonstrated that PFO potentiated the lethal effect of ε toxin in a mouse model for type D enterotoxaemia. This disease naturally occurs predominantly in sheep and goats and is characterized by a rapid proliferation of *C. perfringens* type D strains in the intestine, leading to toxemia and death, sometimes preceded by neurological symptoms [15,120]. ε toxin is generally accepted as the causative toxin for type D enterotoxaemia, since it is the most lethal toxin of *Clostridium perfringens*, and intravenous inoculation of ε toxin reproduces lesions comparable to natural cases [15,120,121,122]. Recently, Garcia *et al.* [117] confirmed the essential role of ε toxin in type D enterotoxaemia, as the mutation of the ε toxin gene in a virulent type D strain eliminated the lethal effect and clinical signs in an intraduodenal inoculation model in mice, sheep and goats. Most type D strains also produce α toxin and PFO. Therefore, Fernandez-Miyakawa *et al.* [8] evaluated the possible synergistic effect of both on ε toxin. Intravenous and intragastric injection of ε toxin in combination with PFO reduced the survival time of mice. For α toxin, a reduction in survival time was only observed after intravenous injection [8]. These results show that the lethal effect of ε toxin can be enhanced by PFO and α toxin.

## 8. Remaining Questions

Cholesterol is essential for pore formation for all cholesterol-dependent cytolysins. Despite the presence of the cholesterol recognition motif, or CRM, in all known CDCs, the timing and role of cholesterol binding can differ between the different CDCs. For PFO, cholesterol seems to be the membrane binding receptor, since the recognition of cholesterol by the CRM in the membrane is the first step in membrane binding. However, the interaction of ILY with cholesterol only occurs after ILY binding to huCD59, with cholesterol then serving to anchor the ILY pore to the membrane following huCD59 disengagement. Whether non-CD59 restricted CDCs utilize an additional receptor is unclear; one study has implicated glycans as a possible membrane receptor, but the data remain uncertain because of the high concentration of competitor glycan required to inhibit CDC binding to cholesterol [123].

Additionally, it is still not clear if sphingolipid- and cholesterol-rich liquid ordered membrane domains or lipid rafts are more prone to cytolysin binding, since the results of completed studies to date are contradictory and inconclusive.

Some gaps remain in our knowledge of the genetic regulation, as well. As already mentioned, there is no insight yet into the signaling molecules and the mode of action of the VirR/VirS system. In addition, the regulatory role of the upstream *pfoR* gene still needs to be clarified.

In spite of widespread PFO production among *C. perfringens* strains, the role of PFO has been underinvestigated in disease development. Future research should take into account the potential contribution of PFO in the pathogenesis of *C. perfringens*-induced diseases, with particular attention on the possible synergistic effect of PFO on the causative toxins. Furthermore, all potentially involved organs should be considered, as the effects can be target organ dependent. Moreover, it is also important that the early events in the disease development are investigated, as PFO might have a significant role in the onset of the disease, whereas its contribution at later stages may be less important.
